# Aluminum Intoxication in Chronic Kidney Disease

**DOI:** 10.1590/2175-8239-JBN-2021-S110

**Published:** 2021-12-03

**Authors:** Rodrigo Bueno de Oliveira, Fellype Carvalho Barreto, Lucas Acatauassu Nunes, Melani Ribeiro Custódio

**Affiliations:** 1 Universidade Estadual de Campinas, Faculdade de Ciências Médicas, Departamento de Clínica Médica, Serviço de Nefrologia, Campinas, SP, Brasil.; 2 Universidade Federal do Paraná, Complexo do Hospital de Clínicas, Serviço de Nefrologia, Curitiba, PR, Brasil.; 3 Universidade Federal do Pará, Belém, PA, Brasil.; 4 Universidade de São Paulo, Faculdade de Medicina, São Paulo, SP, Brasil.

## 1. Diagnosis of aluminum intoxication

1.1 The diagnosis of aluminum (Al) intoxication should be based on clinical suspicion and subsequent laboratory and/or histological confirmation (Evidence) 

1.2 The laboratory diagnosis of Al intoxication is confirmed by the presence of elevated serum Al levels (> 100 µg/L) or a positive desferrioxamine (DFO) test (Al post - Al pre DFO ≥ 50 µg/L) (Evidence).

1.3 To consider the DFO test as false positive associated with the context of serum ferritin levels < 100 ng/mL, or false negative if > 500 ng/mL (Opinion).

1.4 If the DFO test could not be performed, or when a false negative result is suspected, bone biopsy is indicated (Opinion).

1.4.1 Values greater than 30% of the trabecular surface covered by Al are the gold standard for the diagnosis of bone intoxication by this metal (Evidence).

1.5 Al overload in patients with CKD G5D should be monitored every 6 months, by serum dosage or DFO test, when indicated (Opinion).

## 2. Treatment of aluminum intoxication

2.1 Treatment of Al intoxication regardless of the dialysis modality should be done with DFO mesylate, at a single dose of 5 mg/kg/week, intravenously, for 30 to 60 minutes, over a period of 6 months (Evidence). 

2.1.1 For hemodialysis patients, DFO should be administered after the end of the first or second hemodialysis session of the week (Opinion).

2.1.1.1 For patients with serum Al levels > 100 µg/L, DFO should be administered 5 hours prior to the start of the first hemodialysis session of the week (Opinion).

2.1.2 For patients undergoing peritoneal dialysis, DFO should be administered intravenously, with an empty abdominal cavity (Opinion).

2.1.2.1 On automated peritoneal dialysis (APD), DFO should be administered 5 hours prior to the start of dialysis (Opinion).

2.1.2.2 On CAPD, dialysis should only be restarted after a minimum of 5 hours after the end of DFO administration (Opinion).

2.2. DFO should be discontinued in case of serious adverse events, such as visual and/or hearing disorders, drug-attributed allergy, or opportunistic infections (Evidence).

2.3. One month after the 6-month treatment cycle, a new DFO test should be performed to assess the therapeutic response. If the result remains positive, the patient should receive a new treatment cycle (Opinion). 

## 3. Prevention of aluminum intoxication

3.1 Prevention of Al intoxication is done by reducing the patient's exposure to known sources of Al (Evidence).

3.2. Al-based phosphate binders should be proscribed for patients with CKD G3-5D (Evidence).

3.3 The prescription of drugs containing Al in the composition, especially intravenous formulations, should be avoided (Opinion).

3.4 The Al concentration in the water treated for hemodialysis should be lower than 3 µg/L (Evidence). 

## RATIONAL

The toxic effects of aluminum (Al) in patients with advanced CKD, expressed by severe encephalopathy and bone disease, were described more than 45 years ago^1^ ^,^ ^2^. With the efforts to reduce exposure of CKD patients to Al, such as controlling Al levels in the water for dialysis and replacing Al-based phosphate binders by calcium salts, a significant reduction of intoxication cases has been observed^3^. Currently, many authors believe that this condition is limited to sporadic dialysate contamination, inadvertent use of Al-based phosphate binders, or sporadic environmental exposures^4^ ^,^ ^5^. 

While the finding of patients with evident symptoms of Al intoxication has become an uncommon event, the accumulation of Al in bone tissue seems to be an event that is still frequent in our setting, with national data revealing a high prevalence over the last decades^6^ ^-^ ^8^.

A multicenter study identified 2,507 bone biopsies from patients with clinical, radiological or laboratory signs of bone disease. Al intoxication prevalence of 61.3% between 1985-1990; 38.7% between 1991-1996; and 42.4% between 1997-2001 were diagnosed. Among bone tissue samples from Uruguay, a high prevalence of Al intoxication (42% to 27%) was also observed over the decades^6^.

The Brazilian Registry of Bone Biopsies (Rebrabo) analyzed clinical, laboratory and histological data from 260 CKD patients from August 2015 to March 2018, monitored for an average period of 21 months. In a total of 171 available bone tissue samples, 65 (38%) patients had more than 30% of the surface of the bone trabeculae covered with Al. The authors observed no significant differences in the prevalence of Al intoxication among the different types of renal osteodystrophy, or in clinical outcomes such as bone fractures, hospitalization, or death^7^.One possible interpretation is that outcomes captured in short- or medium-term follow-up studies might be impacted by the mitigation of clinical manifestations.

It is known that Al accumulation leads to hematopoiesis and bone mineralization disorders. For example, Rao et al. have observed lower response to erythropoietin treatment in 18 hemodialysis patients with high Al deposition at the mineralization front^9^. Al may also accumulate in the parathyroid glands and interfere with calcium receptors activity and with parathyroid hormone synthesis and release, with consequences on bone tissue^10^ ^,^ ^11^.

Due to this evidence^6^ ^-^ ^11^, it is suggested that the accumulation of Al in bone tissue of CKD patients undergoing dialysis should be actively investigated in our setting at every 6 months. However, the role of routinely applied tests (desferrioxamine test, bone tissue biopsy, and isolated serum Al measurement) remains unclear, for example, in those patients with small elevations in serum Al levels^12^ ^,^ ^13^.

In the Rebrabo study, the diagnosis of Al intoxication was an unexpected finding in about half of the cases. The female gender, prior parathyroidectomy, and hemodialysis treatment were found to be independent predictors of Al accumulation in bone tissue, although the clinical suspicion of Al accumulation by the nephrologist resulted in a poor diagnostic test (sensitivity, 54%; specificity, 65%)^7^. The predictors "parathyroidectomy" and "hemodialysis treatment" could be interpreted as longer CKD and higher exposure to Al-containing sources, respectively.

Classically, the clinical suspicion of Al intoxication should be raised by the presence of proximal myopathy, bone pain, penguin-like waddling gait and fractures, particularly if there is a history of metal exposure. An American study has assessed the frequency of abnormal detection of serum Al levels in thousands of dialysis patients over three years. The authors have observed that only 2.1% to 2.5% of the samples were not within the normal range. It is believed that, despite not reflecting well the chronic exposure to Al or the tissue burden, serum levels could be more useful in the context of recent exposure or in those patients with clinical manifestations of intoxication^12^ ^,^ ^14^.

Kausz et al. have studied the relationship between plasma Al levels and Al accumulation-related bone disease in asymptomatic dialysis patients. Using a plasma value above 40 µg/L as a reference, the authors observed a sensitivity of 65.2% and a specificity of 76.7% for the diagnosis of adynamic bone disease related to Al accumulation^13^. Although Al accumulation in bone tissue has historically been related to low turnover bone disease (osteomalacia and adynamic bone disease), recent data from the Rebrabo study revealed the accumulation of Al in all types of renal osteodystrophy^7^. 

DFO is a drug that has been used for the diagnosis and treatment of Al intoxication since 1980^15^. It has a high affinity for iron and Al, mobilizing these metals from tissue deposits and transferrin. DFO binds to Al resulting in the water-soluble compound called aluminoxamine (C_25_H_45_AlN_6_O_8_; molecular weight = 584.6 g/mol), which is removable through biological and artificial membranes, such as the peritoneal membrane and capillaries for hemodialysis^16^ ^-^ ^18^.

The use of DFO as a diagnostic test for Al intoxication may be performed in case of clinical suspicion of intoxication, acute or chronic exposure to Al sources and prior to parathyroidectomy. The test consists of two fasting blood collections, to determine the serum Al levels, with the 1^st^ and 2^nd^ collections being performed prior to the 1^st^ and 2^nd^ hemodialysis sessions of the week, respectively. After completion of the 1^st^ hemodialysis session, intravenously infuse DFO (5 mg/kg, diluted in 100 mL of 5% glucose solution or 0.9% saline solution, for 60 minutes). The test result is considered "positive" when the difference (delta) in serum Al concentration between the two dosages is > 50 µg/L^19^ ^,^ ^20^. For peritoneal dialysis patients, the DFO test should also be performed with two blood collections separated by a 5-hour interval, with empty abdominal cavity^21^. 

The DFO test, interpreted according to serum parathyroid hormone levels and iron stores, has good sensitivity and specificity in diagnosing Al intoxication^19^ ^,^ ^22^. A positive test combined with a serum parathyroid hormone level < 150 pg/mL has a positive predictive value of 80% for Al-related bone disease, whereas a positive test combined with parathyroid hormone < 650 pg/mL has a sensitivity of 92% and specificity of 86%^20^. False-positive results may be due to sample contamination by Al, therefore a dry tube free of this metal should be used and graphite furnace atomic absorption spectrophotometry, the method of choice. False negative results might happen in dialysis patients with iron overload. The DFO test should be considered false-positive if serum ferritin levels are < 100 ng/mL, or false-negative if > 500 ng/mL^19^ ^,^ ^20^. Therefore, in patients with a strong clinical suspicion of Al intoxication and a negative DFO test, bone biopsy is recommended^19^.

Bone tissue biopsy stained by solochrome azurine and Perls to exclude the presence of iron deposits is the gold standard for the diagnosis of Al intoxication^19^ ^,^ ^23^ ^,^ ^24^_._ However, the cut-off value for the percentage of trabecular bone surface covered by Al in the diagnosis of Al intoxication is controversial, ranging from > 0% to >20%^19^ ^,^ ^20^. In Brazil, specialists and researchers consider as a diagnostic criterion the presence of 30% or more of the trabecular bone surface covered by Al^19^.

The recommended dose of DFO for the treatment of Al intoxication is 5 mg/kg once a week, at the end of the first hemodialysis session of the week, for a period ranging from 3 months to 1 year, a dose which is similar in effectiveness to higher doses, with the advantage of being associated with fewer side effects^25^ ^-^ ^28^. In patients undergoing peritoneal dialysis, DFO can be administered intravenously or intraperitoneally, at the same dose and frequency recommended for hemodialysis patients^19^. The intravenous infusion should be performed slowly, over 60 minutes, with an empty peritoneal cavity. Dialysis should only be restarted after a minimum of 5 hours after the end of the drug administration. DFO is generally well tolerated, but not without side effects. Several studies have reported retinopathy, ototoxicity, dose-related acute neurotoxicity, exacerbation of Al encephalopathy, anaphylactic reactions, and increased susceptibility to opportunistic infections, especially mucormycosis and *Yersinia enteroclitica* ^29^ ^-^ ^31^. Ferrioxamine is a nutrient for microorganisms that use iron in their metabolism. It has been experimentally observed that the presence of ferrioxamine increases the proliferation rate of *Rhizopus* and reduces the therapeutic efficacy of amphotericin B^32^. During treatment with DFO, exacerbation of secondary hyperparathyroidism might be observed due to the removal of Al from multiple tissues in the body, mainly parathyroid and bone^33^ ^,^ ^34^. Treatment control may be managed by means of DFO test or bone biopsy^35^ ^-^ ^37^.

The prevention of Al intoxication consists of measures to reduce patient exposure to Al sources, such as not prescribing Al-based phosphate binders in patients with moderate or advanced CKD or undergoing dialysis, avoiding the prescription of aluminum-containing drugs, especially in intravenous formulations, and maintaining a low Al concentration in the water treated for hemodialysis (< 3 µg/L). 

Drugs and foods contaminated in the preparation process are possible sources of Al exposure. Drugs often prescribed for dialysis patients (i. e., dipyrone, erythropoietin, and iron sulfate), might contain Al, especially in the intravenous formulation^38^. The impact of this contamination is unknown. With respect to diet and Al absorption by the gastrointestinal tract, data on healthy individuals reveal that small amounts (0.06-0.1%) are absorbed from food sources. The factors that may influence the absorption and its bioavailability revolve around compounds that bind to Al in the intestinal lumen, gastric acidity and hardness of the water consumed. Patients with celiac disease might have increased intestinal permeability to Al and thus develop Al-related bone disease^39^ ^-^ ^41^.

Finally, studies have shown that even with current water treatment systems for hemodialysis, small and frequent exposures to Al might be common^42^ ^-^ ^45^. In several countries, including Brazil, the acceptable safety limit for Al concentration in the water for hemodialysis is 10 µg/L (in Brazil, the frequency of this control is every six months)^46^. Health authorities recommend that the acceptable level should be reduced to less than 2-3 µg/L, and with more frequent water quality controls^42^ ^-^ ^45^.
